# Age-Related Neurodegeneration and Memory Loss in Down Syndrome

**DOI:** 10.1155/2012/463909

**Published:** 2012-03-20

**Authors:** Jason P. Lockrow, Ashley M. Fortress, Ann-Charlotte E. Granholm

**Affiliations:** ^1^Department of Neurosciences, Medical University of South Carolina, 173 Ashley Avenue, Charleston, SC 29425, USA; ^2^Center on Aging, Medical University of South Carolina, 173 Ashley Avenue, Charleston, SC 29425, USA

## Abstract

Down syndrome (DS) is a condition where a complete or segmental chromosome 21 trisomy causes variable intellectual disability, and progressive memory loss and neurodegeneration with age. Many research groups have examined development of the brain in DS individuals, but studies on age-related changes should also be considered, with the increased lifespan observed in DS. DS leads to pathological hallmarks of Alzheimer's disease (AD) by 40 or 50 years of age. Progressive age-related memory deficits occurring in both AD and in DS have been connected to degeneration of several neuronal populations, but mechanisms are not fully elucidated. Inflammation and oxidative stress are early events in DS pathology, and focusing on these pathways may lead to development of successful intervention strategies for AD associated with DS. Here we discuss recent findings and potential treatment avenues regarding development of AD neuropathology and memory loss in DS.

## 1. Introduction

The most common cause of dementia is Alzheimer's disease (AD), with rates of prevalence increasing steadily from 60 years of age to reach almost 40% by the age of 85 [1]. AD is defined as the presence of neuritic plaques, which are composed of extracellular deposits of amyloid beta, and neurofibrillary tangles [2]. Neurodegeneration in the later stages of AD is widespread, with massive synapse loss and an overall decline in grey matter resulting from neuronal loss in cortical and hippocampal regions. Cortical neuronal loss is preceded by degeneration of certain subcortical neuronal populations, including basal forebrain cholinergic neurons (BFCNs) [3] and noradrenergic neurons of the locus coeruleus (LC-NE) [4, 5].

While the majority of AD cases are considered sporadic, mutations in amyloid precursor protein (APP) and presenilins 1 and 2 (PS-1 and PS-2) genes are responsible for most of the cases of AD considered “familial” [6]. These mutations lead to alterations in APP metabolism that result in an overabundance of amyloid plaques. Similarly, APP processing is also affected in Down syndrome (DS), a population who exhibit histopathology consistent with AD by the 4th and 5th decades of life with near uniformity, as well as increased risk for dementia [7, 8]. Located on chromosome 21, APP is triplicated in DS, and amyloid-beta deposition is frequently profound in these individuals [9–11]. Recently, cases of familial AD resulting from duplication of only the *APP* locus have been discovered [12], further defining a role for APP in AD dementia. However, few studies have been able to correlate plaque load with dementia severity. Rather, cognitive function correlates most strongly with the degeneration of cholinergic neurons in the basal forebrain. Reversal of cholinergic hypofunction in AD with choline acetyl transferase inhibitors has been shown to facilitate memory function, albeit to a moderate degree [13]. However, it is still not known what causes the cholinergic degeneration, or if other parallel factors also contribute to the disease. Some potential mechanisms include neuroinflammation, oxidative stress, amyloid toxicity, and abnormal phosphorylation of proteins including the microfilament-associated protein tau; etiological causes include genetic mutations, diet, sedentary lifestyle, and environmental toxins [14]. While familial causes of AD are rare and idiopathic AD is difficult to model, DS presents a large and relatively homogenous population with relevant animal models that can serve to illuminate possible etiologies or treatment paradigms in AD.

 In the current paper, we will discuss current theories regarding biological mechanisms and potential treatment paradigms for DS individuals with AD-like dementia (DSD). We include data from animal models, as well as from humans with DSD, and propose potential early prevention models for this difficult and progressive condition.

## 2. Down Syndrome: A Genetic Insight into AD

The uniformity with which individuals with DS acquire AD neuropathology makes this population important to study, not only to gain a better understanding of AD, but also because there are currently no effective treatment paradigms for DSD [8, 15]. Because they have physiological alterations in cardiac and metabolic systems, cholinesterase inhibitors may be contraindicated in some DSD patients [15, 16]. DS is the most common aneuploidy, occurring as frequently as approximately 1 in every 700 live births in the US [17]. DS results in variable levels of intellectual disability, along with congenital defects, and increased risk of certain cancers, such as leukemias [18]. As maternal age continues to increase and medical interventions have increased the lifespan of DS individuals, the prevalence of DSD continues to grow. The diverse and heterogeneous neurodegeneration in AD and in normal aging are accelerated in DS, and lessons learned from DSD patients may uncover therapeutic targets with widespread implications. In fact, DS can be considered a form of segmental progeroid syndrome, or accelerated aging [19, 20].

**Figure 1:**
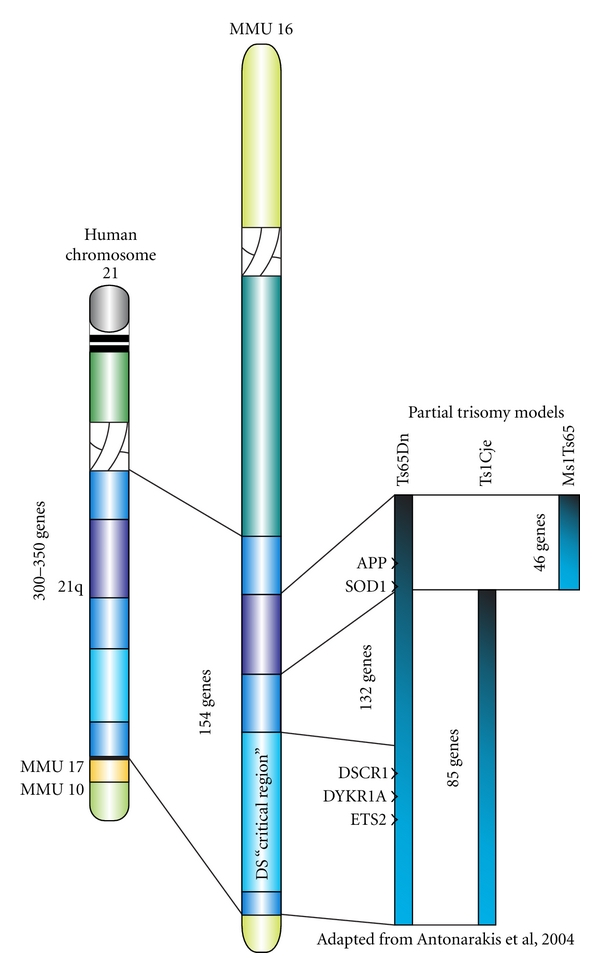
Mouse models for DS. Schematic of the gene segments involved in the so-called “Down syndrome critical region” (DSCR) in human chromosome 21, as well as in different mouse models of the condition. Note that the Ts65Dn mouse contains all genes included in the DSCR, as well as a set of 132 other genes including *SOD* and *APP*. Modified from Antonarakis et al. 2004 [21].

Studies assessing the effects of age on cognition in DS demonstrate a greater incidence of short-term memory impairment in DS individuals over 35 years of age, as well as increasing rates of dementia, aphasia, and agnosia [23] while detriments in executive function are evident already in adolescence [24]. As in idiopathic AD, DSD patients display dysfunction of language and motor skills, seizure onset, and behavioral abnormalities [25], in addition to AD-like pathology, including amyloid-beta deposits, neurofibrillary tangles, loss of BFCNs, and pathological alterations in mitochondria and endosomes [26–29]. While trisomy 21 constitutes the triplication of over 300 genes [30, 31], recent animal studies have sought to elucidate which genes may contribute to the observed neurodegenerative pathology. Based on genetic studies in mouse models of DS, several specific genes contained within the triplicated region of murine chromosome 16 (which corresponds to an equivalent section on human Chr. 21; see Figure 1) have been implicated in the DSD neuropathology. One of the most important genes associated with DS is the *amyloid precursor protein (APP)* gene—increased APP production may partially contribute to DSD-related oxidative stress as well as inflammation. Accumulation of amyloid-beta monomers can directly impair mitochondrial function resulting in energy depletion [32], and it is also well known that accumulation of amyloid—either in tissue culture or *in vivo*—leads to activation of inflammatory cascades [33, 34], most likely via both microvascular dysfunction and activation of resident glial cells in brain parenchyma. Furthermore, cortical DS neurons exhibit impaired mitochondrial function that results in reduced energy production and elevations in reactive oxygen species (ROS) [35]. Studies using the Ts1Cje mouse model for DS, which does not include triplication of the SOD or APP genes [36], suggest that other triplicated genes may be involved in mitochondrial abnormalities observed in DS. In addition, while *APP* and *SOD-1* each may contribute to the disease, neither gene is solely responsible for the degenerative changes that occur in DS [37]. Other genes located on the critical region include Ets-2 and DSCR1 (Figure 1), which have both been linked to neurodegeneration [35, 38]. In this paper, we will provide evidence, from our recent work and others, suggesting that inflammation and oxidative stress are early dysregulations which may be responsible for age-related dementia and associated pathology in DSD.

## 3. Modeling DS Pathology: The Ts65Dn Mouse

**Figure 2:**
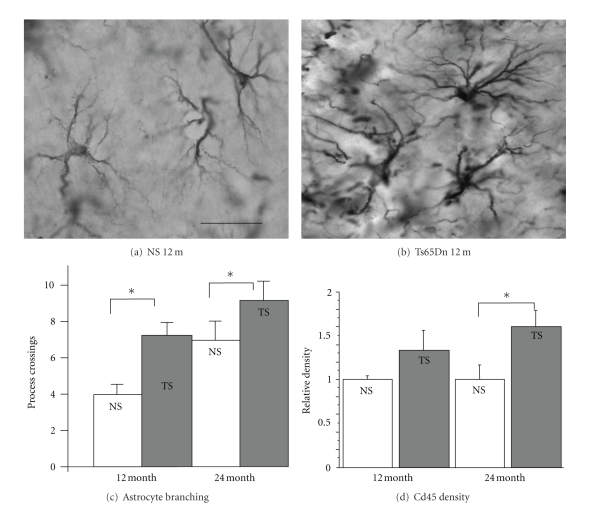
Gliosis in hippocampus of Ts65Dn mice. Brain tissue sections from Ts65Dn mice and age-matched normosomic littermates, showing typical hippocampal morphology of astrocytes, using the glial marker GFAP (a) and (b). The sections were from 12-month-old normosomic (NS, a) or Ts65Dn (TS, b) mice. Note increased number of astrocytes in TS mice, as well as elevated expression of GFAP and an activated morphology, with more branching and thicker branches in the TS compared to NS mouse. (c) Astrocyte branching measurements (GFAP labeling) in the hippocampus reveal increased branching in TS mice compared to NS age-matched controls, a sign of activation following inflammatory or other pathological processes. Astrocytosis is increased with aging in the TS mice to a greater extent than in NS mice. (d) Density of a marker for microglial cells, Cd45, is also increased with age in Ts65Dn (TS) but not in age-matched normosomic (NS) mice, indicating ongoing microglial activation in this brain region. Inset in (b) represent 100 microns. *Data were not published previously*.

As discussed elsewhere in this issue, a spontaneous translocation of a portion of murine chromosome 16 onto chromosome 17 led to the formation of a DS model, the Ts65Dn mouse [39]. The translocated segment of chromosome 16, syntenic to a significant portion of human chromosome 21 (Figure 1), thus provided a genetic triplication which can be passed on to offspring [39]. Nearly 140 known genes are triplicated in Ts65Dn mice, of which 60% are also located on human chromosome 21 [40]. More importantly, these mice exhibit normal lifespans, allowing for the analysis of progressive neurodegenerative alterations. While Ts65Dn mice fail to develop amyloid plaques, they do exhibit elevated levels of APP and associated peptides in the hippocampus [41–43] and increased phosphorylation of tau protein [44, 45]. Ts65Dn mice also show increased inflammatory morphology with aging [22, 46] (see also Figure 2) synaptic dysfunction [47, 48], and a failure of neurotrophic signaling, particularly involving the retrograde transport of nerve growth factor (NGF) to the basal forebrain [42, 46, 49, 50], and downregulation of brain-derived neurotrophic growth factor (BDNF) levels [51, 52]. In addition, they exhibit age-related degeneration of LC-NE and BFCN neurons [22, 53–55]. Memory deficits are progressive in these mice and onset coincides with BFCN atrophy [43, 46, 56]. Interestingly, a study by Belichenko et al. [57] suggested that 33 genes, included in the so-called “DS critical region” (DSCR) of genes in humans, and triplicated in a novel mouse model (Ts1Rhr), might be responsible for many of the physiological and behavioral detriments observed in the Ts65Dn mice, narrowing the search for the set of genes involved in DSD neuropathology [57]. However, other studies have shown that although this “critical region” is necessary for cognitive impairment and pathology to develop [58], overexpression of these particular genes is not sufficient to generate DSD, at least not in mouse models, demonstrating the complex nature of DS-related dementia and neuropathology with aging.

While degeneration of basal forebrain cholinergic neurons (BFCNs) occurs during normal aging, DSD and AD are defined by rapidly accelerated loss of these projection neurons, and cholinergic dysfunction correlates strongly with the progression of cognitive decline in both diseases [59, 60]. Ts65Dn mice show consistent learning and memory deficits on spatial reference and working memory tasks [56, 61–67]. Most of these deficits become apparent between 4 and 12 months of age [56], suggesting, indeed, that the behavioral dysfunction developing in the Ts65Dn mouse mimics the segmental progeria syndrome observed in terms of brain function in humans with DS. Ts65Dn mice exhibit deficits in novel object tasks, which are reversed by the partial *N*-Methyl-D-aspartic acid (NMDA) glutamate receptor blocker Memantine (Namenda) [68–70]. These findings suggest that glutamate and GABA transmitter systems are affected by the genetic alterations in Ts65Dn, directly or indirectly, in Ts65Dn mice, something that has been suggested by work from other research groups as well [71, 72]. In a manuscript by Rueda et al. [71], they found that treatment with memantine in aged Ts65Dn mice improved spatial learning but did not affect the number of dentate granule cells, suggesting that the effects of memantine may be pharmacological, rather than neuroprotective. These data were further supported by our findings, that memantine increased working memory performance, particularly in a novel object task, but did not rescue hippocampal, cholinergic, or locus coeruleus neurons from progressive neurodegeneration [70]. The cognitive impairment observed over time in Ts65Dn mice parallels cognitive impairment in adult DS individuals with early or moderate AD, tested on the WISC-R behavioral battery, showing progressive deterioration in executive function, comprehension, picture completion, vocabulary, and digit span [73]. The memory deficits indicate hippocampal and frontal cortex dysfunction and together with septohippocampal degeneration indicate that the Ts65Dn mouse is a unique model to understand the progression of neuropathology and memory loss in DSD.

## 4. Locus Coeruleus Degeneration in DSD

**Figure 3:**
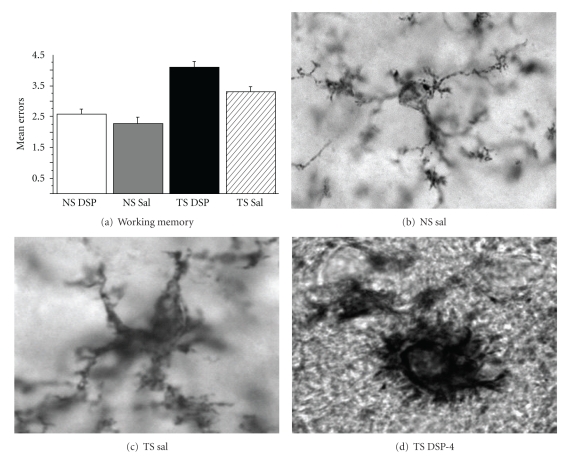
Effects of the NE neurotoxin DSP-4 on Ts65Dn and normosomic mice. Note significant aggravation of performance in a memory task (a) coupled with aggravated activation of microglial cells (b–d) in the hippocampal formation, as evidenced by Cd45 immunohistochemistry. (a) Average number of errors in a water radial arm maze. The NE lesion exhibited more pronounced effects on errors in the maze in TS than in NS mice, and TS mice performed more errors than NS mice, regardless of NE lesions (DSP) or not (Sal). (b–d) Cd45 staining of microglial cells in the hippocampus in a normosomic mouse (NS) treated with saline (b), a Ts65Dn mice on saline (c), and a Ts65Dn mouse that received DSP-4 lesions of the LC-NE neurons (d). Note significant activation of individual microglial cells as a result of the NE lesion in TS mice compared to controls. Quantitation of inflammatory processes is available in Lockrow et al., 2011 [22].

LC-NE degeneration, while less studied than BFCN loss, is another hallmark of AD [74]. NE neurotransmission exerts effects on neurons, glia, and blood vessels throughout the neuraxis. LC-NE lesions, using the selective NE neurotoxin DSP-4, give rise to aggravated amyloid accumulation, oxidative stress, and memory loss in transgenic AD models [75–77]. Findings suggest that LC-NE effects are mediated both directly, via neurotransmission changes in the limbic system, and indirectly, via aggravation of amyloid accumulation, inflammation, and oxidative stress pathways. NE-mediated neuroprotection of oxidative stress on BCFNs *in vitro* is independent of adrenergic receptor activation or intracellular accumulation, [78] suggesting a role for NE in the neutralization of hydroxyl radicals. The antioxidant activity of NE provides a pharmacological link between LC-NE and cholinergic survival. NE circuitry also exhibits a direct influence on memory formation. BFCNs activity is modulated by NE via adrenergic receptor activity [79], and pharmacological stimulation of NE receptors leads to improved cognitive performance both in rodent models and in humans [80]. While NE is an essential modulator of memory through its ability to regulate synaptic mechanisms, NE depletion is not sufficient to significantly alter memory function in intact animals [22]. Yet, NE depletion in the presence of cholinergic dysfunction exacerbates memory impairments [22] and may therefore aggravate deficits in memory systems dependent on the basal forebrain cholinergic neurons. In a recent study, Ts65Dn and NS mice were lesioned using the NE neurotoxin DSP-4 at 4 months of age and were then studied at 8–10 months of age in terms of behavior and neurochemistry. As can be seen in Figure 3 and in [22], the NE lesion gave rise to a significant aggravation of both memory loss and neuropathology in Ts65Dn but not in NS mice, including degeneration of hippocampal and BFCNs as well as increased inflammatory markers. These findings suggest that NE neurotransmission, albeit important for normal function of the brain, plays a particularly important role for curbing age-related pathology in the form of inflammation and neuronal loss. This notion has been supported by other investigators, showing enhanced effects of DSP-4 lesions in APP transgenic mice [76, 81, 82]. These investigators also found that administration of the NE precursor L-threo-DOPS restored microglial functions in NE-depleted mice [76], suggesting a reciprocal system where the amyloid cascade, inflammatory markers, and NE innervation systems affect each other. Interestingly, others have also shown that LC neurons spontaneously degenerate in AD mouse models [83], again suggesting a specific link between accelerated amyloid accumulation and degeneration of LC neurons.

Importantly, individuals with DSD exhibit early and progressive degeneration of LC-NE neurons [84]. Recently, a study by Salehi et al. [55] demonstrated successful recovery from memory loss in Ts65Dn mice using the NE precursor Droxidopa (L-threo-dihydroxyphenylserine). These results are promising and should be considered in future clinical treatment paradigms for DSD patients. Since LC-NE degeneration is common to both Parkinson's disease (PD) and AD patients [85–87], future pharmaceutical interventions for dementia may include enhancement of NE neurotransmission also for these neurological conditions. Promising clinical pilot studies have already been initiated in terms of the NE reuptake inhibitor Atomoxetine and memory loss in PD [88] and in Alzheimer's disease [89, 90] even though much remains to be done in terms of incorporating NE enhancement treatment for dementia. LC-NE neurons partake in the regulation of blood vessels, microglial cells, as well as neurons, and degeneration of this monoaminergic cell group can be an active player in neuropathological processes in age-related dementia of different etiology.

## 5. Inflammatory Pathology in AD and DSD

As in AD, individuals with DSD consistently exhibit chronic inflammation in limbic system areas of the brain, with increases in microglial and astrocytic activation coupled with IL-1*β* and TNF-*α* cytokine release [91–93]. Microglial activation typically arises in the entorhinal cortex before developing in the hippocampus and surrounding cortex as well as the basal forebrain [26, 27]. BFCNs are highly sensitive to inflammation and oxidative stress [94], but specific biological mechanisms for their selective loss in AD and in DSD have not been revealed. There is also evidence that TNF-*α*-induced cortical inflammation at cholinergic terminals leads to retrograde degeneration of BFCNs [95]. Recent work suggests that inflammation due to loss of noradrenergic innervation from the LC-NE innervation of BFCNs is a plausible explanation for the selective vulnerability of these neurons in DSD and AD [22]. *β*-adrenergic receptors are expressed in astrocytes and microglia and modulate the cytokine release [96]. The reduction of noradrenergic neurons in the LC correlates with amyloid plaques and dementia severity in AD [97, 98]. NE treatment of cholinergic cells *in vitro *reduces expression of IL-1*β* and TNF-*α*, as well as proinflammatory proteins such as iNOS [96]. Since Ts65Dn mice exhibit significant degeneration of both BFCNs and LC-NE neurons, it is not surprising that we found accelerated and age-related astrocytosis and microgliosis in the hippocampus of this mouse model of DS (Figures 2 and 3). As mentioned above, depletion of noradrenergic terminals in murine models of AD results in increased inflammatory cytokine production, activated microglial morphology, and amyloid deposition [76, 82, 99]. NE terminal destruction also impeded cholinergic neurotransmission in AD models which otherwise show no cholinergic deficits [81]. Thus, while inflammation may affect many of these neurodegenerative processes, it also can increase in response to early abnormalities in ACh and NE signaling, since there is a reciprocal relationship between neuronal and glial modulation of inflammatory processes, especially during neurodegenerative disease [96]. Based on these studies, it is difficult to determine whether BFCN and LC-NE degeneration activates the inflammatory pathways, or if the cytokine production by astrocytes and microglia, in turn, causes the neuronal degeneration in DSD and AD. Most likely, all of these processes have interactive and escalating effects on each other, leading, in the end, to memory loss and AD pathology.

## 6. Neurotrophic Factors and DS

The survival and maintenance of BFCNs depend on neurotrophic support from NGF and BDNF [100]. NGF mRNA is expressed at high levels in regions innervated by cholinergic terminals, such as the neocortex, dentate gyrus, and the hippocampal pyramidal layer [3]. Upon release from postsynaptic neurons, NGF binds to its high-affinity receptor, TrkA, on BFCN nerve terminals, initiating receptor oligomerization which leads to signaling cascades through PI3K and ERK activation and endocytosis of the ligand-receptor complex [101]. This complex is retrogradely transported to the soma where it facilitates signal transduction of phenotypic markers such as choline acetyltransferase [101, 102]. Exogenous administration of NGF rescues BFCNs from age- or toxin-related degeneration and reverses cognitive dysfunction in animal models of AD or normal aging [103]. While the production of NGF in the hippocampus and cerebral cortex has been shown to be unaltered or even increased in AD [103], NGF levels in the basal forebrain exhibit significant decline [104]. A compensatory increase in NGF expression in target regions may be due in part to loss of TrkA receptor expression in BFCN neurons, which occurs early in AD and is recapitulated in aged rodents [3, 105]. Murine models for DS show reductions in retrograde NGF trafficking which occurs in part due to enlarged, dysfunctional endosomes [42, 49, 101]. Recent studies have shown that these endosomal changes can be caused by overexpression of APP [42, 106]. Abnormal endosomes are present in both AD and DSD brains [29] and localize to the vulnerable regions such as the basal forebrain and the hippocampus [107] suggesting that endosomal trafficking of NGF linked to TrkA may be a pathological pathway to explore further in DSD brains.

BDNF also promotes BFCN survival and cholinergic signaling [108–110]. BDNF expression is reduced in AD [109], and BDNF levels are reduced in serum from DS individuals [111], and in brain tissue from the Ts65Dn mouse model for DS [52], and has been shown to be linked to memory function, as well as synaptic plasticity and neurogenesis [112]. BDNF expression is increased following exercise and may therefore contribute to the beneficial effects of voluntary exercise observed in AD as well as in normal aging in humans and animal models [113–117]. Interestingly, several studies have shown that LC-NE innervation into cortical regions regulates the expression of BDNF, suggesting a close link between loss of BDNF expression and LC-NE degeneration in DS [118]. In a recent manuscript by Counts and Mufson [119], the authors demonstrated that administration of NE protected cultured neurons from amyloid-beta-mediated toxicity by upregulating both NGF and BDNF expression. Further, the authors found that NE inhibited increased reactive oxygen species (ROS) and caspase activation caused by the neurotoxin, suggesting also a direct link between the neurotrophic factors, NE innervation, and oxidative stress. Treatment with functional blocking agents for NGF and BDNF removed the beneficial effects, indeed suggesting that NE effects were mediated by the trophic factors. This paper therefore linked several pathological processes in DSD and AD, providing direction for future research and treatment options. Our recent study using Ts65Dn mice extended these findings *in vivo*, by showing that an LC-NE lesion, using the neurotoxin DSP-4, decreased BDNF expression in frontal cortex, a region associated with working memory loss in the Ts65Dn mouse model [22]. We also found a significant correlation between BDNF expression and NE levels, as well as between BDNF expression and working memory errors, suggesting a clear link between BDNF expression and memory function dependent on this region. BDNF and NGF have been associated with neuroprotection against oxidative stress in neurons [119, 120], suggesting that DSD patients may exhibit increased sensitivity to oxidative stress because of reduced expression of these neurotrophic factors.

## 7. Oxidative Stress and DSD Pathology

Individuals with DS exhibit elevated oxidative stress early in life [121]. Oxidizing free radicals, also known as ROS, are cytotoxic byproducts of normal mitochondrial metabolism and are normally processed by endogenous antioxidants. But when levels of mitochondrial ROS production exceed the intracellular antioxidant defenses, oxidative molecules can disrupt cellular functions, negatively affecting synaptic plasticity and eventually leading to neuronal injury and apoptosis [122]. The hippocampal formation exhibits a high vulnerability to both ischemic and neurotoxic injury associated with oxidative stress [123]. A marker of RNA oxidative damage, 8-hydroxyguanosine (8-OHG), is elevated in neurons of the hippocampus and cortex early in the progression of AD and precedes much of the pathology in these regions, suggesting that oxidative stress may be the earliest event in AD-related disease processes [124]. Postmortem analysis revealed that 8-OHG immunoreactivity increased significantly in cortical neurons of DS individuals in their teens and twenties, while amyloid-beta burden was increased only after 30 years of age [125], strongly suggesting that oxidative stress is an early event also in DS. The central question is why is oxidative stress so rampant in the brain of DS individuals?

**Figure 4:**
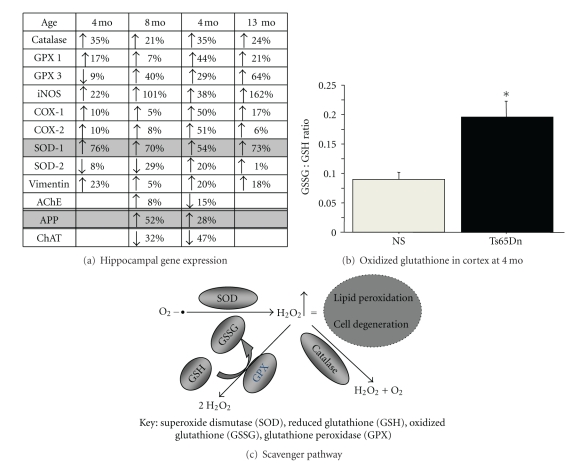
(a) Superarray (SABiosciences, Frederick, MD) against oxidative stress and inflammatory markers was used on hippocampal tissue from pooled samples (3 per group) of Ts65Dn and Normosomic mice at 4, 8, 10, and 13 months of age. Note the increased expression in *APP* and *SOD-1* due to increased gene dosage of these genes. However, glutathione peroxidase 1 and 3 (GPX 1 and 3), as well as catalase levels, were not increased to the same extent. Further investigation of the glutathione enzymatic pathway revealed increased GSSG:GSH ratio in Ts65Dn compared to normosomic brain (b), suggesting, a compensatory processing of free radicals, but not sufficient to eliminate peroxidation in neurons. Glutathione exists in two forms: GSH (reduced form) and GSSG (oxidized form). Normally the relationship between these two forms is 1 : 10 in healthy cells. (c) Schematic representation of the ROS scavengers, demonstrating that elevated SOD levels may lead to increased H_2_O_2_ levels, leading to enhanced stress on the glutathione and catalase pathways. *Data were not published previously. *

Part of the answer to that question may be the triplication of both *APP* and *SOD-1* genes in DS (Figure 1). The balance between ROS production and the scavenger enzyme pathways is tightly regulated in the cell during normal conditions. We propose that the increase in expression of SOD-1 in DS leads to a reduction in superoxide but an increase in the accumulation of hydrogen peroxide (H_2_O_2_) in tissues. This hypothesis is based on a superarray using pooled samples of tissue from the hippocampus of Ts65Dn mice revealing significant elevations in hippocampal SOD-1 expression with only a moderate increase in the other scavenger enzymes, including glutathione reductase and catalase (Figure 4). Elevated rates of conversion from superoxide to H_2_O_2_ would lead to lipid peroxidation in neurons and glia, accumulating with time, and leading to the neuropathology observed in Ts65Dn mice with age, as well as in DS individuals. This hypothesis was recently validated by studies from Harris-Cerruti et al. [37], showing that a mouse model consisting of double SOD-1/APP overexpression leads to memory loss and neuropathology, as well as elevated ROS in the brain, while APP overexpression alone was less effective in generating neurodegeneration or ROS accumulation. When the investigators examined hippocampal slices for long-term potentiation (LTP), they found that LTP was impaired in both tg-SOD and tg-APP-SOD mice, but not in tg-APP mice, suggesting that the APP overexpression alone did not affect this cellular component of hippocampal plasticity. SOD-1 overexpression alone also gave rise to ROS accumulation, but not to the extent observed in APP/SOD-1 overexpression mice, suggesting a comodulation of oxidative stress pathways by the *APP* and *SOD-1* genetic overexpression [37].

There is a controversy in the literature regarding beneficial or damaging effects of SOD overexpression. While some investigators show that SOD-1 or SOD-2 overexpression rescues neuropathology in AD transgenic mouse models [126], others demonstrate aggravated pathology when over-expressing SOD-1 [126], suggesting that there is a complicated relationship between SOD-1 and SOD-2 function in the CNS. Gardner and colleagues [127] investigated this question using a minimal mathematical model. The authors concluded that the outcome depended on a balance between processes consuming superoxide without forming H_2_O_2_ and those consuming superoxide with high H_2_O_2_ yield [127]. Our investigations shed some light on this particular question for DS brains, since Ts65Dn mice exhibited elevated expression of both glutathione and catalase (Figure 4), presumably as a response to elevated H_2_O_2_ levels in the brain. However, since most investigators use indirect methods of measuring H_2_O_2_, such as measuring lipid peroxidation or associated markers, it has not been shown, at least not to our knowledge, whether neurons or glia from DSD patients or Ts65Dn mice exhibit elevated H_2_O_2_ levels, even though studies of postmortem brain tissue have shown that levels of peroxiredoxin, which is an enzyme involved in eliminating H_2_O_2_, are elevated in both DSD and AD [128]. The role of oxidative stress in development of pathology in DS individuals is further discussed in other sections of this issue.

Early increases in ROS suggest that antioxidant therapy may benefit DS individuals with AD pathology. While clinical results for vitamin E treatment in AD patients have been mixed to this point [129], there have been minimal studies to determine whether antioxidants could be beneficial in DSD, despite a recent study of vitamin E administration during childhood in DS [130]. We recently reported beneficial effects of long-term vitamin E treatment in Ts65Dn mice [131] and suggest that this may be a viable future option for DSD. Ts65Dn mice were given vitamin E in their diet from 4–10 months of age, and cognitive performance was tested, followed by brain pathology. BFCN and hippocampal cell loss were reduced significantly, and neuroinflammation associated with microglial activation was also significantly reduced, suggesting a strong connection between inflammatory and oxidative stress pathways [131]. Oxidative stress measures correlated with improved cognitive performance, supporting the hypothesis that oxidative stress plays an important role for memory loss associated with DSD. Based on these encouraging findings, and the relatively minor risks associated with vitamin E treatment, we would suggest future development of this treatment paradigm for individuals with DS as a prevention strategy.

## 8. Overexpression of APP: Disease Modifier

An involvement of the amyloid cascade in the progressive memory loss and neuropathology in DS cannot be denied. It is likely that the overproduction of APP in DS individuals (Figure 1) converges upon both oxidative stress and inflammation pathways in the brain, to cause added harm to the DSD patient with time. Amyloid-beta-induced oxidative stress appears to be mediated through an NMDA receptor-mediated increase in Ca^2+^ influx [132]. Elevated intracellular Ca^2+^ disrupts mitochondrial function [133] and may explain the reduced mitochondrial efficiency seen in AD. As previously shown by our laboratory, Ts65Dn mice have deficits in expression of calbindin, a neuronal calcium-binding protein, in the hippocampus [46], suggesting further dysregulation of intracellular Ca^2+^ pathways. It is also possible that other genetic components of the triplicated gene segment aggravate DS-related AD pathology. The regulator of calcineurin 1 (RCAN1 or DSCR1) is also over-expressed in DS and in Ts65Dn mice (Figure 1). A recent manuscript by Porta et al. [134] demonstrated that RCAN1 knockout neurons (RCAN1^−/−^) exhibited a reduced response to oxidative stress, and the investigators therefore suggested vulnerability to oxidative stress downstream from the SOD-1-mediated accumulation of H_2_O_2_ in DS and in AD. These findings are important for continued efforts in determining the role of different genes in DS to provide additional substrates for neuroprotection strategies.

## 9. Outstanding Questions

Outstanding questions in this field should focus on prevention and/or treatment options for DSD. As individuals with DS live longer and medical interventions have been able to modify cardiovascular problems or other health issues, the incidence of DSD will go up dramatically in the next couple of decades. Based on recent findings related to vitamin E and antioxidant capacity, we feel that it is important to assess prevention in DS individuals at an early stage using vitamin E and/or other antioxidants. Further, treatment with NE enhancing drugs, such as Atomoxetine (Strattera) [88, 135], has shown promising results in children with ADHD and in PD; it is possible that these pharmaceutical interventions may be beneficial for working memory deficits and early onset problems with executive function in persons with DSD as well. It is important to note that several disease processes, related to inflammation, oxidative stress, cholinergic cell loss, calcium homeostasis, amyloid accumulation, and locus coeruleus degeneration, all converge on the progressive deficits observed in the limbic system of individuals with DS with age. Combination therapy targeting several aspects, or working upstream from the observed pathology, should therefore be developed. Finally, a national registry for DSD and age-matched control brain tissue and associated tissues is long overdue. The development of such a repository will allow centralized and streamlined studies into etiology but also possible treatment paradigms for DSD and finally render this field well-deserved attention, using a nation-wide collaboration for DSD-related studies.
